# The Status of Psychological Issues Among Frontline Health Workers Confronting the Coronavirus Disease 2019 Pandemic

**DOI:** 10.3389/fpubh.2020.00265

**Published:** 2020-06-05

**Authors:** Zeming Hu, Bin Chen

**Affiliations:** ^1^Department of General Surgery, The First Affiliated Hospital of Gannan Medical University, Ganzhou, China; ^2^Key Laboratory of Prevention and Treatment of Cardiovascular and Cerebrovascular Diseases, Ministry of Education, Gannan Medical University, Ganzhou, China

**Keywords:** COVID-19, health workers, medical staff, infection, mental health, psychological intervention

## Introduction

In late December 2019, an outbreak of a pneumonia caused by novel coronavirus disease 2019 (COVID-19) infection was reported in Wuhan, Hubei province, China, which has since spread domestically and internationally (1). According to a report by The World Health Organization (WHO), as of May 22, 2020, 4,995,996 cases of COVID-19 infection have been confirmed globally using specific laboratory RT-PCR (2). Among these cases, 84,520 were from China, 228,006 from Italy, 129,341 from Iran, and 1,525,186 from the USA. Most of the infected patients are admitted to designated hospitals for systemic treatment and isolation. This has resulted in unprecedented psychological distress and other mental health symptoms among frontline health workers worldwide engaged in the fight against the COVID-19 pandemic (3).

## Psychological Impacts

In *the Lancet*, unfortunately, it was reported that 16 healthcare workers were infected at a stage when the transmissibility of COVID-19 was not well-defined (4, 5). As an increasing number of studies about the transmission routes of severe respiratory syndrome coronavirus 2 (SARS-CoV-2) have been conducted, healthcare workers who come into direct contact with confirmed or suspected patients are at high risk of infection despite the use of personal protective equipment (PPE). According to the National Health Commission of China, more than 3,300 medical professionals have been infected with COVID-19. In Italy, as of April 16, 2020, 16,991 healthcare providers who handled confirmed patients had been infected, and 127 physicians died (6). This implies that medical staff, especially those at the frontline in the fight against the pandemic without sufficient PPE or other essential equipment, are likely to fear for their own safety and that of their close friends, colleagues, and even families. Infected health workers confirmed COVID-19 patients potentially causing a negative feeling of frustration and helplessness. Healthcare workers are therefore under tremendous mental health stress during the ongoing COVID-19 crisis (7).

Prevailing evidence indicates that elderly patients complicated with chronic diseases or common comorbidities are susceptible to acute respiratory distress syndrome (ARDS), acute respiratory failure, and multiple organ failure among other conditions (5). With no specific and effective antiviral drugs or vaccines, patients infected with COVID-19 are seemingly staring death in the eye. Such patients are primarily given symptomatic treatment to relieve severe clinical manifestations with the help of breathing machines. Effective communication with patients and relatives is compromised by the use of PPE, which covers most of the face. This challenging situation makes health professionals feel guilty, helpless, and depressed, which eventually results in common mental disorders such as anxiety, depressive disorders, and post-traumatic stress disorder (PTSD) (8). As the COVID-19 outbreak continues to spread, many suspected infections or close-contact visits to designated hospitals increase the workload and number of working hours for healthcare providers. This leads to emotional strain and physical exhaustion.

## Importance of Protecting Mental Health

The critical situation mentioned above is a reminder of previous infectious disease outbreaks. Healthcare providers who participated in the fight against the previous 2003 SARS outbreak have experienced a broad range of psychological problems, including stress, depression, and anxiety, some of which have persisted for several months after the outbreak (9). Research from the H1N1 influenza epidemic shows that many healthcare workers developed symptoms of PTSD, depression, anxiety, and burnout within a few weeks of the outbreak (10). This is consistent with a recent psychological survey that demonstrated that the odds of developing depression, anxiety, stress, and insomnia symptoms among health professionals working in the designated hospitals are 50.7, 44.7, 73.4, and 36.1%, respectively (11). Another recent survey from China indicated that a considerable proportion of medical staff who participated in the epidemic prevention and control reported symptoms of depression (50.4%), anxiety (44.6%), distress (71.5%), and insomnia (34%) (12). Therefore, effective strategies to subvert mental breakdown among medical providers are needed as part of the public health response to the ongoing COVID-19 pandemic.

## Psychological Intervention Strategies

In this opinion piece, we highlight the utility of psychological services and support systems for healthcare workers participating in the control of COVID-19 pandemic. Strategies and initiatives employed by the Chinese Health Authorities to handle the psychological issues among frontline health workers during the early stage of the COVID-19 epidemic as well as the lessons learnt are discussed. The Chinese government has set up multidisciplinary mental health teams, including the psychosocial response team, psychological intervention technical support team, psychological intervention medical team, and psychological assistance hotline team, all of which are mandated to implement preparedness strategies to reduce the negative psychological impact of COVID-19 on medical providers (8). The strategies utilized include telephone-, internet-, and application-based counseling and intervention by online platforms. The WHO and many other institutions have designed guidelines to provide psychological support for medical staff during the current pandemic outbreak. For instance, the WHO has released a 30-point guideline for mitigating the developing psychological issues among healthcare workers (13). The guideline highlights the need for medical professionals to protect themselves, their family members, friends, and colleagues accordingly.

In addition to the social support systems provided by organizations, building proper self-awareness, peer support, and team support will equip medical workers with the capacity to cope with mental health stress during the current pandemic. A smooth relationship between healthcare workers and COVID-19 patients should be established (14). Healthcare workers should work as team to avoid burnout (15). Mechanisms for effective communication should be put in place to allow health care workers update their leaders about their working conditions and schedule for break from work (16). During treatment, medical professionals should ensure that each treatment procedure is effective, understand the availability of medical resources, and learn to establish self-confidence (17). Medical workers should have enough sleep since inadequate sleep and high workloads may weaken the immune system (17). Thus, hospitals should provide essential services such as a place to rest, food, daily living supplies, avenues for communication with families to alleviate anxiety, and sufficient PPE (18). This will improve the psychological well-being of medical staff.

The importance of peer and team support from colleagues or teams should not be underestimated. Peer groups share common experiences through shorthand ways known to all members. Members of the peer group communicate freely without the fear of breaking taboos as their social rules have been established. Talking to co-workers who may be conversant with the experiences in the working environment is an approach with which we can control emotional stress during this pandemic (19). Furthermore, teams need to encourage each other and find approaches to assist new members feel safe, valued, and welcome as quickly as possible. Constant encouragement, cheering, and affirmation of each other will improve the treatment outcomes. Team members should not blame each other, and, in case of mistakes, solutions should be developed in a timely manner. Observance of these factors will undoubtedly improve the capacity of healthcare workers to cope with the immense psychological pressure during the on-going COVID-19 pandemic (20).

## Summary

The safety and mental health of first-line medical workers must be closely monitored during the fight against a pandemic. Frontline health workers need effective support to help them cope with arising mental health problems. First, Health Authorities worldwide must implement strategies to address problems such as high workloads, hospital supplies, hospital beds, among others. Second, social support, including online services and guidelines provided by organizations, should be utilized to timely, effectively, and efficiently mitigate the psychological impacts among health workers. Third, proper self-awareness, peer support, and team support are encouraged as part of healthcare system response in the context of public health emergency. Healthcare workers should prioritize their own well-being as much as possible, addressing their essential needs for food, rest, and sleep and understanding the treatments they can afford. In addition, the feasibility and effectiveness of communication and encouragement within groups or teams should be suggested to minimize the detrimental consequences during the COVID-19 pandemic. The timely address of psychological crisis among medical workers preferably based on the above strategies is important.

## Author Contributions

ZH drafted and revised the manuscript. BC reviewed the manuscript for approval. All authors agreed the final version.

## Conflict of Interest

The authors declare that the research was conducted in the absence of any commercial or financial relationships that could be construed as a potential conflict of interest.

## References

[1] Wang C Horby P Hayden FG Gao GF. A novel coronavirus outbreak of global health concern. Lancet. (2020) 395:470–3. 10.1016/S0140-6736(20)30185-9 31986257 PMC7135038

[2] World Health Organization (WHO) Novel Coronavirus (SARS-CoV-2) Situation Reports. (2020). Available online at: https://www.who.int/docs/default-source/coronaviruse/situation-reports/20200522-covid-19-sitrep-123.pdf

[3] Greenberg N Docherty M Gnanapragasam S Wessely S. Managing mental health challenges faced by healthcare workers during covid-19 pandemic. BMJ. (2020) 368:m1211. 10.1136/bmj.m1211 32217624

[4] Huang C Wang Y Li X Ren L Zhao J Hu Y. Clinical features of patients infected with 2019 novel coronavirus in Wuhan, China. Lancet. (2020) 395:497–506. 10.1016/S0140-6736(20)30183-5 31986264 PMC7159299

[5] Chen N Zhou M Dong X Qu J Gong F Han Y. Epidemiological and clinical characteristics of 99 cases of 2019 novel coronavirus pneumonia in Wuhan, China: a descriptive study. Lancet. (2020) 395:507–13. 10.1016/S0140-6736(20)30211-7 32007143 PMC7135076

[6] de Girolamo G Cerveri G Clerici M Monzani E Spinogatti F Starace F. Mental health in the coronavirus disease 2019 emergency—the Italian response. JAMA Psychiatry. (2020) 77:E1–3. 10.1001/jamapsychiatry.2020.1276 32352480

[7] Xiang Y Yang Y Li W Zhang L Zhang Q Cheung T. Timely mental health care for the 2019 novel coronavirus outbreak is urgently needed. Lancet Psychiatry. (2020) 7:228–9. 10.1016/S2215-0366(20)30046-8 32032543 PMC7128153

[8] Kang L Li Y Hu S Chen M Yang C Yang BX. The mental health of medical workers in Wuhan, China dealing with the 2019 novel coronavirus. Lancet Psychiatry. (2020) 7:e14. 10.1016/S2215-0366(20)30047-X 32035030 PMC7129673

[9] Maunder RG Lancee WJ Balderson KE Bennett J Borgundvaag B Evans SL. Long-term psychological and occupational effects of providing hospital healthcare during SARS outbreak. Emerg Infect Dis. (2006) 12:1924–32. 10.3201/eid1212.060584 17326946 PMC3291360

[10] Mcalonan GM Lee AM Cheung V Cheung C Tsang KWT Sham PC. Immediate and sustained psychological impact of an emerging infectious disease outbreak on health care workers. Canad J Psychiatry. (2007) 52:241–7. 10.1177/070674370705200406 17500305

[11] Liu S Yang L Zhang C Xiang Y Liu Z Hu S. Online mental health services in China during the COVID-19 outbreak. Lancet Psychiatry. (2020) 7:E17–8. 10.1016/S2215-0366(20)30077-8 32085841 PMC7129099

[12] Lai J Ma S Wang Y Cai Z Hu J Wei N. Factors associated with mental health outcomes among health care workers exposed to coronavirus disease 2019. JAMA Netw Open. (2020) 3:e203976. 10.1001/jamanetworkopen.2020.3976 32202646 PMC7090843

[13] World Health Organization (WHO) Mental Health and Psychosocial Considerations during the COVID-19 Outbreak. (2020). Available online at: https://www.who.int/docs/default-source/coronaviruse/mental-health-considerations.pdf

[14] Pan YT Wang H Chen SR Zhang C Research on the strategy of solving the psychological crisis intervention dilemma of medical staff in epidemic prevention and control. Chin Med Ethics. (2020) 3:1–5.

[15] Maben J Bridges J. Covid-19: supporting nurses' psychological and mental health. J Clin Nurs. (2020). 10.1111/jocn.15307. [Epub ahead of print]. 32320509 PMC7264545

[16] Walton M Murray E Christian MD. Mental health care for medical staff and affiliated healthcare workers during the COVID-19 pandemic. Eur Heart J Acute Cardiovasc Care. (2020) 9:241–7. 10.1177/2048872620922795 32342698 PMC7189614

[17] Wu W Zhang Y Wang P Zhang L Wang G Lei G. Psychological stress of medical staffs during outbreak of COVID-19 and adjustment strategy. J Med Virol. (2020). 10.1002/jmv.25914. [Epub ahead of print]. 32314806 PMC7264502

[18] Chen Q Liang M Li Y Guo J Fei D Wang L. Mental health care for medical staff in China during the COVID-19 outbreak. Lancet Psychiatry. (2020) 7:E15–6. 10.1016/S2215-0366(20)30078-X 32085839 PMC7129426

[19] Teoh K Kinman G Looking after doctors' mental wellbeing during the covid-19 pandemic. BMJ opinion. (2020). Available online at: https://blogs.bmj.com/bmj/2020/03/26/looking-after-doctors-mental-wellbeing-during-the-covid-19-pandemic/

[20] Brooks SK Webster RK Smith L Woodland L Wessely S Greenberg N. The psychological impact of quarantine and how to reduce it: rapid review of the evidence. Lancet. (2020) 395:912–20. 10.1016/S0140-6736(20)30460-8 32112714 PMC7158942

